# Paradoxical Exploitation of Protected Fishes As Bait for Anglers: Evaluating the Lamprey Bait Market in Europe and Developing Sustainable and Ethical Solutions

**DOI:** 10.1371/journal.pone.0099617

**Published:** 2014-06-17

**Authors:** William L. Foulds, Martyn C. Lucas

**Affiliations:** School of Biological and Biomedical Sciences, Durham University, Durham, County Durham, United Kingdom; Pacific Northwest National Laboratory, United States of America

## Abstract

A reoccurring conservation problem is the resolution of consumptive use of threatened wildlife and is especially difficult to defend when it occurs for recreational practices. We explored the commercial capture and supply of threatened European river lamprey (*Lampetra fluviatilis*) to anglers, to determine the extent of exploitation and seek opportunities for improved conservation. The trade began in 1995 from England, but by 2012 involved sale of lamprey from England, The Netherlands and Estonia, including from protected populations. Lamprey are sold frozen for the capture of predatory fish, mostly in freshwater. In the year 2011/2012 9 tonnes (>90,000 lampreys) of river lamprey were supplied, almost exclusively to British anglers. Although annual catches in the main English lamprey fishery (River Ouse) have varied widely since 1995, catch per unit effort did not decline between 2000 and 2012. Conservation actions since 2011 have included a cap on fishing licenses, catch quotas and restricted fishing seasons. Now, 86% of lamprey bait is imported to Britain. Most bait sellers interviewed would not stock lamprey if they knew they were from threatened populations; many felt their trade would not be impacted if lamprey were not stocked. This facilitates opportunities to enter into dialogue with anglers over alternative baits to threatened lamprey. The study emphasises the need to inform stakeholders about conservation species subjected to market-driven exploitation.

## Introduction

Conflicts often exist between the agendas of conservationists and stakeholders who are involved in the consumptive use of a wildlife resource, particularly if the resource is threatened [1]–[5]. Whilst the consumptive use of a threatened wildlife resource is perhaps socially defensible under certain conditions (e.g. to offer nutrition or to support local development), it may be difficult for stakeholders to rationalise when the resource is harvested for recreational purposes (e.g. angling, sport hunting, pet trade). It is therefore important to examine, from both an ecological and socio-economical perspective, instances in which the exploitation of a threatened species has legal legitimacy and is driven by the demands of recreational users. Such circumstances tend to be characterised by different stakeholder attitudes towards regulation and improved conservation outcomes are likely to require consensual negotiation [2], [6]–[8].

The worldwide exploitation of lampreys, of which over half of species are threatened [9]–[11], typifies some of the conflicts between conservation objectives and stakeholder motivations. For instance, both the anadromous river lamprey (*Lampetra fluviatilis*) and sea lamprey (*Petromyzon marinus*) are widely considered threatened in Europe [10]–[13] although globally they are Least Concern according to the International Union of Nature (IUCN) Red List of Threatened Species [14], [15]. They are listed as protected species under Annex III of the Bern Convention and require protection by member states of the European Union under Annex II of the Habitats and Species Directive (92/43/EEC). However, under Annex V of the same directive, their exploitation may be allowed and subject to management measures. They are harvested in several European countries as high-value food [13], [16]–[18]. Although the decline of both species in Europe has been attributed to river regulation, habitat degradation and pollution, exploitation has also represented a threat to their sustainability [10], [19]–[21].

Collection of animals for fishing bait, whether for commercial or recreational purposes, can potentially have substantial conservation implications [22]–[24], requiring careful consideration. In about 1995 a market was developed in Britain for adult European river lamprey, captured during their spawning migration and sold to anglers as bait for predatory fish, especially pike (*Esox lucius*) [25]. This was paralleled by commercial harvesting of river lamprey for bait in the tidal reaches of the River Ouse, which discharges into the Humber estuary, the largest estuary on the east coast of Britain and a designated Special Area of Conservation (SAC) protected area in which this species is a listed feature [25]. At the time, although potential impact on the SAC gave legal recourse for cessation of lamprey exploitation on precautionary grounds, only since 2011 has legal enforcement occurred (see section 1.1.).

How heavily the lamprey bait market in Britain is dependent on catches in the Humber River Basin, and whether the population has declined since the advent of fishing there, is unknown. Just how large is the river lamprey bait market, is it restricted to Britain and are lamprey imported from other European waters to satisfy British anglers? Given that river lamprey are widely considered to be threatened in Europe, there is a pressing need to account for the origin of the lamprey being sold in Britain.

Furthermore, the potential effects of restricting river lamprey harvests on stakeholders’ livelihoods have not been explored. Failing to consider the knowledge and views of stakeholders, when attempting to conserve and regulate the system from which they derive benefits, can ultimately lead to the failure of conservation efforts [7], [26]–[29]. Knowledge gained through investigating (a) how informed stakeholders are about the resource they use, (b) the potential impacts on stakeholders’ livelihoods from regulating the resource, and (c) how amenable stakeholders are to proposed regulations of the resource, can better inform policy-making decisions and help predict the effects of conservation actions [6], [30]–[32].

Here we present the first examination into the river lamprey angling bait (henceforth referred to as lamprey bait) market, including analyses of catches from a protected area, a description of the structure and scale of the lamprey bait market in Britain and the attitudes of key stakeholders. Our findings offer insights for regulators and demonstrate the importance of learning from stakeholders to inform and alert conservationists about potential future conflicts.

### 1.1 History of the Fishery and Legislation

Whilst a commercial fishery for river lamprey operated in the Humber River Basin (including its two main subcatchments, the Ouse and Trent) in the 19^th^ and early 20^th^ centuries for human consumption and to supply bait for the North Sea long-line fishery [25], it collapsed, and only re-emerged in 1995 with the development of an angling market. On the Ouse, Masters et al. [25] found that up to 31,000 adult river lamprey had been harvested by one commercial fisherman each fishing season (typically from October to January) and estimated that the relative exploitation by that fisherman during one season (2003/2004) was 12%. They also discovered other fishermen exploiting the same resource in the Ouse and small numbers of lamprey also taken from the Trent [25].

Humber lamprey were legally caught as by-catch in an authorised European eel (*Anguilla anguilla*) fishery, even though they outnumbered eel in autumn-winter by two orders of magnitude. Lamprey were not recognised as a “freshwater fish” in the fisheries legislation (Salmon and Freshwater Fisheries Act (SAFFA), 1975). Therefore, lamprey fisheries (i.e. number of fishing permits, landings size) could not be legally regulated by that law; nor were landings reports enforceable. The key threat to the Humber SAC river lamprey population was regarded as unregulated fishing exploitation [25]. The UK Marine and Coastal Access Act 2009 amended SAFFA to legislate for lamprey and other diadromous species not covered previously, and from January 2011 enabled licensing of lamprey fisheries, with temporal and total catch restrictions imposed. This aided careful regulation of lamprey exploitation in Britain; in the case of the Humber River Basin it allowed selective entry only of existing lamprey fishermen.

## Methods

Data collected in this study is stored on a securely backed-up system at Durham University. Any requests for access to use data should be made to the corresponding author in the first instance.

### 2.1 Commercial Catch Data

The only significant contemporary river lamprey commercial catch dataset in Britain pertains to lamprey trapped below Naburn weir (53°54′N, 01°06′W) by two separate commercial fishers on the tidal Ouse, between 1995 and 2012. Before 2011, river lamprey were caught mostly between October and January, reflecting the main period of upriver migration there [25]. Since 2011, due to license restrictions, a maximum of 1044 kg lamprey (split equally between the two fishers) can be taken between 1^st^ November and 10^th^ December in the Ouse; an estimated exploitation level of 5%.

Both fishers have used fyke nets and/or unbaited, two-funnel eel traps to catch lamprey [25]. The first fisher (Fisher A) used a combination of nets and traps between fishing seasons 1995 and 1999. In this paper, trapping lamprey from year χ through to year χ+1 is referred to as fishing season χ (e.g. Oct 2000 to Feb 2001 is referred to as the 2000 season). From 2000 onwards Fisher A used traps only. He upgraded ten of his traps from uncovered to black netlon covered in 1999, as he believed they fished more effectively, and had upgraded all of his traps to black netlon covered by 2011. Whether the second fisher (henceforth referred to as Fisher B) has ever altered his fishing gear is unclear. While Fishers A and B use the same 3 km river reach for fishing, they use different sites (one each), the locations of which have remained the same for Fisher A since 2000, and for Fisher B for all data obtained.

Catch data were collected from the fishermen and from the Environment Agency (EA). Although the submission of lamprey catches to the EA has been statutory since 2011, reporting before 2011 was voluntary. Consequently, catch data from the tidal Ouse before 2011 is incomplete. Fisher A provided total catch data (lbs or kg) for 1995–2008, 2011 and 2012, although finer scale information was provided for 2000–2008, 2011 and 2012, with the catch (lbs or kg) and number of traps fished for each date the traps were lifted being recorded. This allowed catch per unit effort (CPUE) to be calculated for each date the traps were lifted as mean weight per trap, and could be converted to mean number of lamprey per trap per day using a mean weight for an individual lamprey of 101.2 g [25]. Although Fisher B could only provide data for 2004, 2005, 2011 and 2012 (though he has fished a similar number of seasons to Fisher A), data was comprehensive, thus both total seasonal catch and CPUE for each date the traps were lifted could be calculated.

The extent to which CPUE varies within fishing seasons was examined and the date in which CPUE is expected to be highest for any given fishing season was estimated. Gaussian curves were independently fitted to CPUE data from all fishing seasons for Fisher A (2000–2008 and 2011–2012) and Fisher B (2004, 2005, 2011, 2012). The expected CPUE on date *t* in season *j* for either fisher was given as:



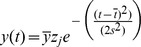
.

Where 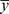 is the maximum CPUE in 2000 season (Fisher A) or 2004 season (Fisher B), 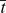 is the day in which CPUE is highest, 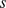 is a measure of the spread in CPUE and 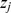 is the relative difference in CPUE from season to season where 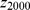 = 1 (Fisher A) or 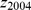  = 1 (Fisher B). The curves were fitted using maximum likelihood assuming the variation in the data about the mean had gamma distributions (see [33]).

### 2.2 Stakeholder Telephone Interviews

#### 2.2.1. Ethics statement

As part of the telephone interview process respondents were assured that answers would remain anonymous and confidential, informed that data may be pooled for publication, and that answering questions was voluntary. Oral consent was given by respondents and was documented electronically. The study, including the oral consent procedure, received ethical approval by Biological and Biomedical Sciences Ethics Sub-Committee, University of Durham.

#### 2.2.2. Interview methodology

The key stakeholders in the river lamprey bait market include the commercial fishers, wholesalers, tackle shop managers and pike anglers. We considered both wholesale suppliers and tackle shop managers, as they constitute easy-to-reach groups, for whom the supply and demand of lamprey in Britain, and the scale and structure of the market, can be determined. Moreover, their businesses may depend strongly on lamprey sales, therefore they are likely to be the most financially impacted by legislation affecting the supply of lamprey.

The telephone questionnaire method was selected because it can yield high response rates and allows views to be expressed in detail. The contact details of interviewees were, in this case, easily accessible online, and allowed collection of data over a wide geographic area [34], [35]. Three separate telephone questionnaires were generated:

1. a questionnaire (up to 26 questions asked) targeted at river lamprey *wholesale supplier* managers in Britain, conducted between 11 December 2012 and 11 January 2013;
2. a questionnaire (up to 50 questions asked) targeted at *tackle shop* managers listed in a major river lamprey wholesale supplier’s directory, conducted between 19 July and 22 August 2012; and.
3. a questionnaire (three questions asked) targeted at *tackle shop* managers listed in a major online telephone directory, conducted between 2 September and 6 November 2012.

The aims of questionnaires one (Q1) and two (Q2) were to understand: the extent and importance of river lamprey sales in their businesses; the impact that lamprey unavailability might have on their businesses; how knowledgeable managers were about the lamprey they sell, in the context of conservation; and whether managers show concern for the conservation status of the lamprey they sell, and whether they would personally alter their ‘selling behaviour’ of lamprey, or agree to a ban on the capture and sale of lamprey in Britain, if they are considered to be threatened. The aim of questionnaire 3 (Q3) was to better determine the extent of lamprey sales in Britain. Combining information would enable determination of the origin of the lamprey sold in Britain, the size and structure of the lamprey bait market in Britain, the number of wholesale suppliers and tackle shops selling lamprey and the number of river lamprey stocked in British tackle shops in a single year, and the occurrence of sales by wholesalers to the angling trade outside Britain.

Wholesale supplier managers, the respondents to Q1, were identified by ‘snowball sampling’, a non-probability sampling procedure which benefits from known members of a population being able to identify ‘hidden’ members of a population [36]. Tackle shop managers from Q1 and Q2 sampling frames, if they sold lamprey, were asked to identify their lamprey supplier(s). Every supplier identified was contacted and asked to confirm that they supplied lamprey. If so, the manager was invited to complete the questionnaire.

A known major wholesaler of lamprey in Britain permitted the use of their online directory to contact tackle shop managers for Q2. The supplier stated that their business supplies lamprey to the majority of their tackle shop customers, therefore sampling from this sampling frame (427 tackle shops) would ensure a high probability of calling tackle shops selling lamprey. The supplier directory sampling frame was stratified by region (Scotland, North England, East England, West England/Wales and South England) and tackle shops were randomly sampled from these. Tackle shops targeted for Q3 were identified using the Yellow Pages online telephone directory (www.yell.com), one of the most comprehensive in Britain. “Fishing Tackle” in “England”, “Wales” and “Scotland” was searched, producing 1,614 tackle shops. A total of 333 *Yellow Pages* shops, duplicated from the supplier’s sampling frame, were removed. Remaining tackle shops were stratified by postcode district (accessed from www.list-logic.co.uk), after which a tackle shop was randomly selected from a randomly selected postcode district and contacted.

Although a brief explanation was provided as to the purpose of the questionnaires, mention of lamprey being considered a threatened species was avoided to prevent biasing respondents’ views. Sensitive questions (defined by Tourangeau and Ting [37] as those that potentially stimulate a socially undesirable response) were asked towards the end of each questionnaire, so as to minimise the risk of early termination of the questionnaire [38].

Both Q1 and Q2 consisted of closed and open questions, and similar questions were asked to allow comparisons between stakeholders. Many closed questions required respondents to agree, disagree or find it difficult to say, to a statement or select a response provided in a Likert scale [39], e.g. “If lamprey were no longer available to sell, would this have 1) no impact, 2) a slight impact, 3) a moderate impact, 4) a strong impact on your business?”. As there are far more tackle shops than wholesale suppliers, most Q2 questions were closed to allow quantitative analysis. Chi square tests of independence or logistic regressions were used to identify variables influencing tackle shop managers’ decisions to continue, or alter, their selling ‘behaviour’ or their agreement, disagreement or indecision regarding a ban on the capture and sale of lamprey in Britain. Potential variables included: how important they felt it was to know if the lamprey they sell are from a threatened or non-threatened population; the number of years the business has sold lamprey; the number of lamprey stocked by the business over a one year period (summer 2011 to summer 2012); how ‘replaceable’ they believed lamprey are as a bait; the impact of lamprey unavailability on the business. Q3 consisted of just three questions: *does your shop sell lamprey*?; *how many lamprey did you sell from summer 2011 to summer 2012*?; and *which supplier(s) supply your lamprey*?

## Results

### 3.1 Commercial Catches in the River Ouse

**Figure 1:**
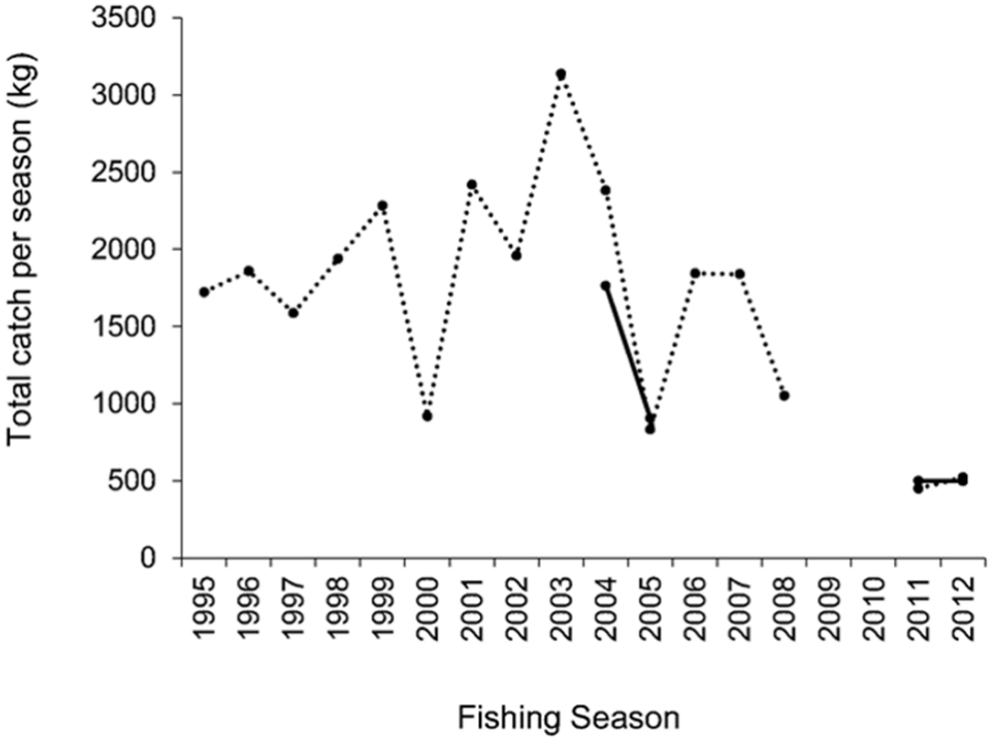
The total catches of river lamprey (in kg) by Fisher A (dotted) and B (solid) for seasons 1995–2008 (lamprey caught as by-catch in licenced eel fishery) and seasons 2011–2012 (lamprey caught in an authorised lamprey fishery with temporal and allowable catch restrictions) in the tidal River Ouse, Humber River Basin, NE England.

Before fishing restrictions in 2011, catch data from 2000–2008 indicate that Ouse lamprey were fished from as early as 9^th^ September (2006 season, Fisher A) up to 21^st^ February (2000 season, Fisher A), and the number of traps and days fished varied between seasons. Before 2011, total catch (kg) of river lamprey caught by Fisher A varied moderately between fishing seasons (1995–2008), ranging from 834.2 kg in 2005 (equivalent to *∼*8 243 lamprey) to 2 810.5 kg (∼30 998 lamprey) in 2003 (Fig. 1). Mean seasonal total catch (kg) for Fisher A for seasons 1995–2008 was 1 841.5±625.8 kg (± SD), equivalent to ∼18 197±6 184 lamprey. Before 2011, total catch (kg) of river lamprey caught by Fisher B ranged from 904.5 kg (∼8 937 lamprey) in 2005, 8.4% more than Fisher A’s total catch for 2005, to 1 764.9 kg (∼17 443 lamprey) in 2004, 25.9% less than Fisher A’s total catch for 2004 (Fig. 1). Thus, mean seasonal total catch (kg) for Fisher B for 2004 and 2005 was 1 334.7±608.4 kg (± SD), equivalent to ∼13 189±6 012 lamprey. The total catch (kg) of lamprey by both fishers varied little in 2011 and 2012 when fishing restrictions were imposed (Fig. 1).

A Gaussian curve was fitted to CPUE data for all fishing season for both fishers. For Fisher A, the model predicts that 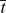 (average peak CPUE) is 14^th^ December, with an expected CPUE of 11.28 lamprey per trap for that day. On average, 68% and 95% of catch for a season is made between 8 November – 9^th^ January (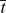 ± σ) and 3^rd^ October – 24^th^ February (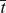 ±2σ), respectively. For Fisher B, 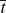 is 8^th^ December, with an expected CPUE of 9.95 lamprey per trap for that day. On average, 68% and 95% of catch for a season is made between 8 November – 7^th^ January (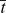 ± σ) and 9^rd^ October – 6^th^ February (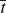 ±2σ), respectively.

**Figure 2:**
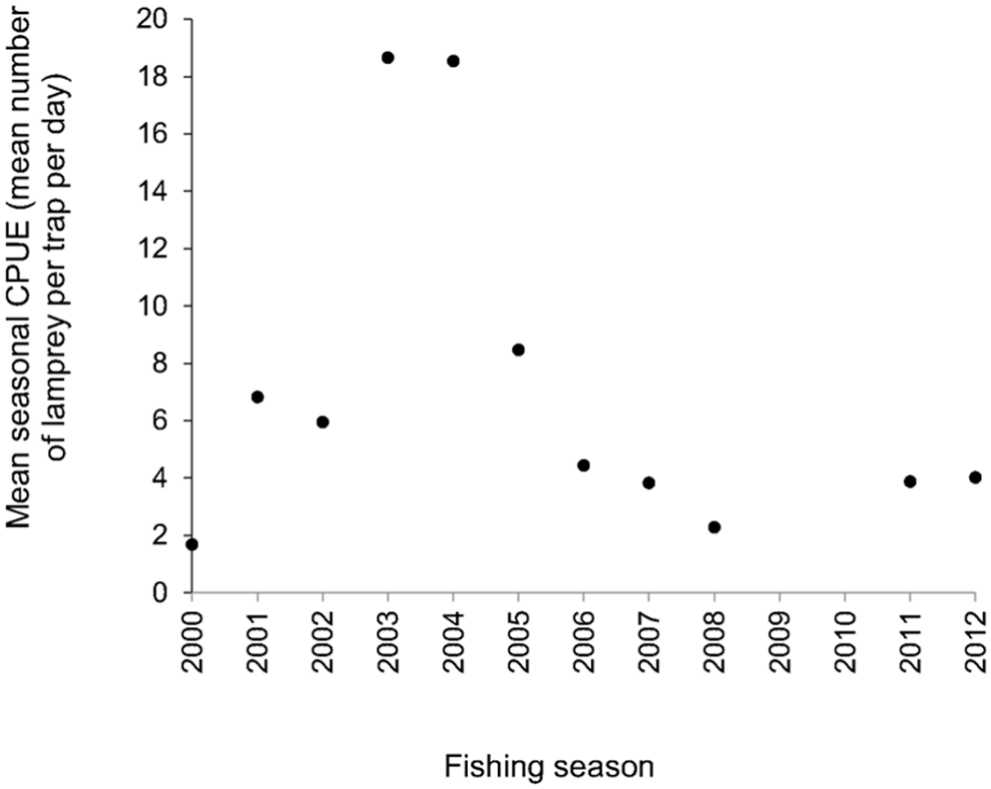
A scatterplot of mean seasonal CPUE (mean number of lamprey per trap per day) against fishing season for Fisher A’s catch data. There was no significant relationship between variables.

For Fisher A’s catch data between 2000–2012 fishing seasons, when only traps at the same site were used, enabling CPUE to be used as an abundance index, the mean CPUE (weighted by number of traps fished) for each season was calculated (referred to henceforth as mean seasonal CPUE). There was no relationship between mean seasonal CPUE and year (Linear regression, *F*_1,9_ = 0.821, *P* = 0.388, *R* ^2^ = 0.084; Fig. 2), hence there was no evidence of a trend in abundance change. Given that the lifecycle of river lamprey requires approximately 4–6 years of larval growth and about 1–2 years of adult growth [40], analysis of 2000–2012 fishing seasons covers year classes originating from just before the start of the fishery, and for a subsequent period of 12 years.

### 3.2 Wholesale Supplier Interviews

**Figure 3:**
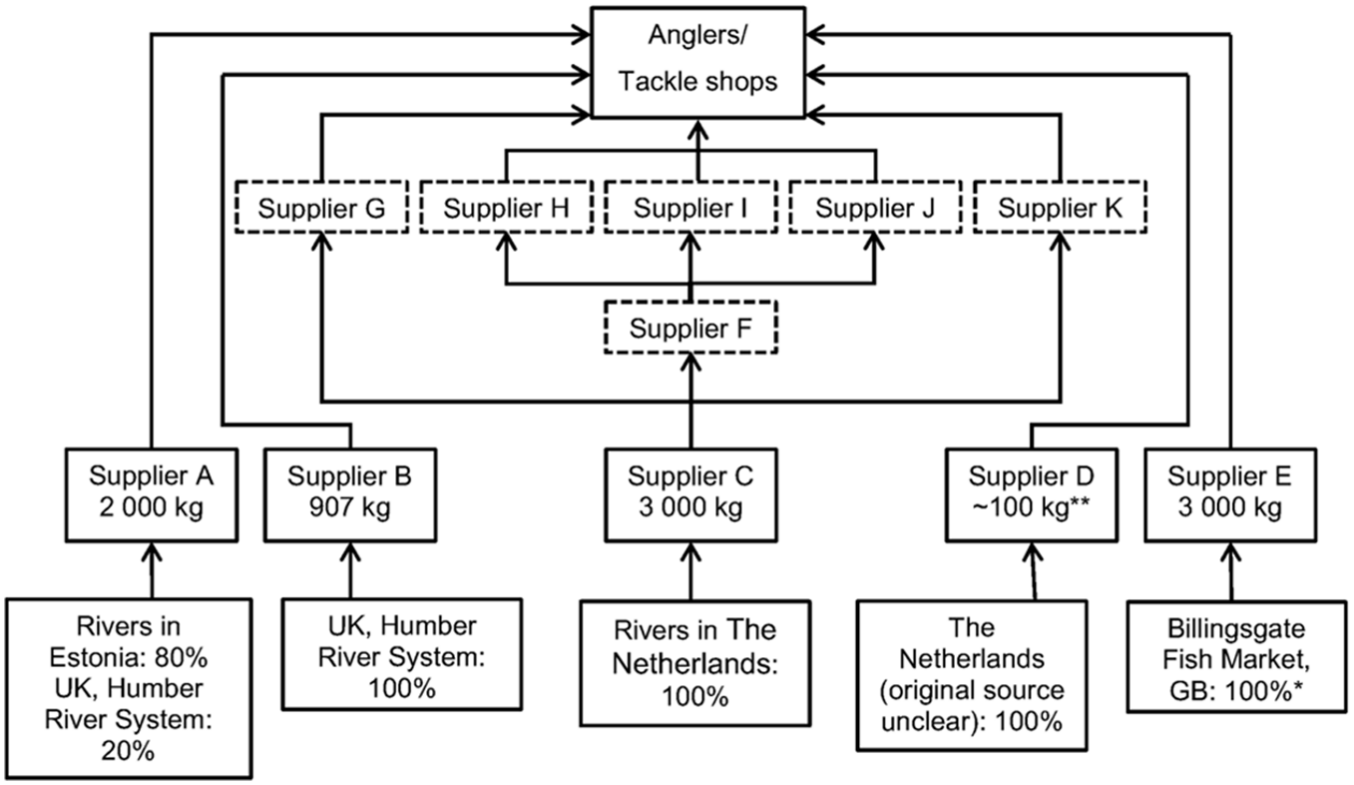
A schematic showing the river lamprey bait market structure in Britain. Suppliers A–E (bold boxes) obtained lamprey from either Britain, The Netherlands or Estonia, whilst suppliers F–K (dashed boxes) obtained lamprey from other suppliers. Suppliers C and F were indirect suppliers of lamprey i.e. only supplied lamprey to other suppliers. Arrows denotes the movement of lamprey products. *Supplier E suggested that lamprey from Billingsgate fish market were sourced from The Netherlands and Britain. **The number of lamprey was provided by Supplier D, which was converted to a weight based on an individual river lamprey weighing on average approximately 100 g from The Netherlands (Lanzing, 1959).

After collective identification by tackle shop managers (Q2, Q3) and successful telephone contact, 12 British wholesale suppliers confirmed they sold river lamprey, of which 11 agreed to participate (Suppliers A–K, Fig. 3). Six of them (Suppliers F–K) obtained lamprey from other suppliers in Britain, five (Suppliers A–E) obtained lamprey directly from fishers operating in the Humber River system, Britain, Billingsgate fish market, London, or they imported lamprey from The Netherlands or Estonia (Fig. 3).

Suppliers’ A–E approximations of the lamprey bait supplied by their business between summers 2011 and 2012, totalled 9.01 tonnes (Fig. 3). Supplier E could only state to the best of their knowledge that the large majority of lamprey from Billingsgate fish market, London, originated from The Netherlands, therefore a cautious total estimate of 6100 kg of lamprey were sourced from this country by Suppliers C–E. Supplier C has been sourcing lamprey from The Netherlands since *c.* 2005. Eighty percent of Supplier A’s lamprey were sourced from Estonia, and this supplier first started importing from Estonia in 2002, at an average rate of about 1500 kg p.a. The river systems supplying lamprey in The Netherlands and Estonia are unknown.

Suppliers A–E answered further questions and were relatively knowledgeable about the lamprey they sell. Four named the lamprey species they sell (all river lamprey), and all were aware of which country they originated from (Fig. 3), however, three were unaware of which river system their lamprey were sourced from. Two suppliers believed the lamprey they sell are from a non-threatened population, whilst one was unsure. The remaining two suppliers understood that river lamprey are of conservation concern but regulations are in place to help protect them, and indeed one supplier has been involved in regulating the fishery from which they source their lamprey. Several wholesalers stated that almost all of their lamprey bait is currently for use in Britain; none were aware of significant markets of lamprey for angling bait elsewhere. The only other non-human food purpose was, for Supplier A, provision to the zoo-aquarium trade as a replacement for the endangered European eel.

Whilst two suppliers claimed there would be no impact on their business if lamprey sold for bait became unavailable, three claimed they would be moderately, strongly and very strongly impacted, respectively. Four disagreed that there are other products to sufficiently replace lamprey, whilst the remaining supplier was unsure. When asked if they would discontinue lamprey supply if they were informed they were from a threatened population, four said yes whilst one declared they would be the first to ensure the fishery was operating in a sustainable way if this was the case. One supplier agreed there should be a ban on the capture and selling of lamprey if they were threatened, one supplier was unable to comment, and the remaining three found it difficult to say. The latter group felt that before they could make an informed decision they would need to be provided with rigorous scientific evidence confirming the lamprey they sourced were threatened.

### 3.3 Tackle Shop Managers Interviews

By independently multiplying the estimated total number of tackle shops in the major suppliers and *Yellow Pages* sampling frames by the median number of lamprey stocked by the tackle shops from summer 2011 to 2012, and summing the figures, we estimated that a total of between 77, 880 and 92, 520 river lamprey were stocked by tackle shops in both sampling frames (Table 1). If an average weight of 100g for lamprey from the Humber [25], The Netherlands [41] and Estonia (Anon. wholesaler, pers. comm.) is taken, this constitutes 7.79–9.25 tonnes of lamprey per annum.

**Table 1:** Estimations of the number of lamprey stocked by British tackle shops from the major suppliers and *Yellow Pages* sampling frames between summer 2011 and summer 2012.

	Major supplier’ssampling frame	*Yellow Pages*sampling frame	
Total no. tackle shops	427	1 281	
Total no. contacted	289	200	
Number that sell lamprey (proportion as %)	251 (86.9)	106 (53.0)	
95% confidence interval of proportion	±3.9%	±6.9%	
Estimated total no. tackle shops that sell lamprey (min–max)	354–387	590–768	
Median no. lamprey stocked by tackle shops for 2011–2012	120	60	**Total**
Estimated no. lamprey stocked 2011–2012 (min–max)	42 480–46 440	35 400–46 080	**77 880–92 520**

Of 251 tackle shops contacted in the major supplier’s sampling frame that sold lamprey bait, 137 (54.6%) completed Q2. A further 60 respondents were willing to complete the survey, but due to time constraints they either partially completed the questionnaire (13.5%) or requested to be contacted again (10.4%), often on multiple occasions. Therefore, a total of 197 respondents (78.5%) were happy to cooperate with the survey. Only 4 respondents terminated the questionnaire partway through, suggesting little response bias. Respondents who sold river lamprey did so as frozen bait, either sectioned or whole, for predatory fish, mostly pike, but also other freshwater species such as catfish (*Silurus glanis*), chub (*Squalius cephalus*) and marine species such as conger eel (*Conger conger*) and mackerel (*Scomber scombrus*)). Just one respondent claimed it was their most popular frozen bait; the other most popular baits were mackerel (*Scomber scombrus*) (40.0% of shops), squid (13.3%), roach (*Rutilus rutilus*) (12.4%) and smelt (*Osmerus eperlanus*) (11.4%). Responses were mixed when asked about the popularity of lamprey over the last five years, with 24.8%, 32.3% and 42.9% of respondents remarking that the popularity of lamprey with their customers had decreased, increased and remained the same, respectively.

**Figure 4:**
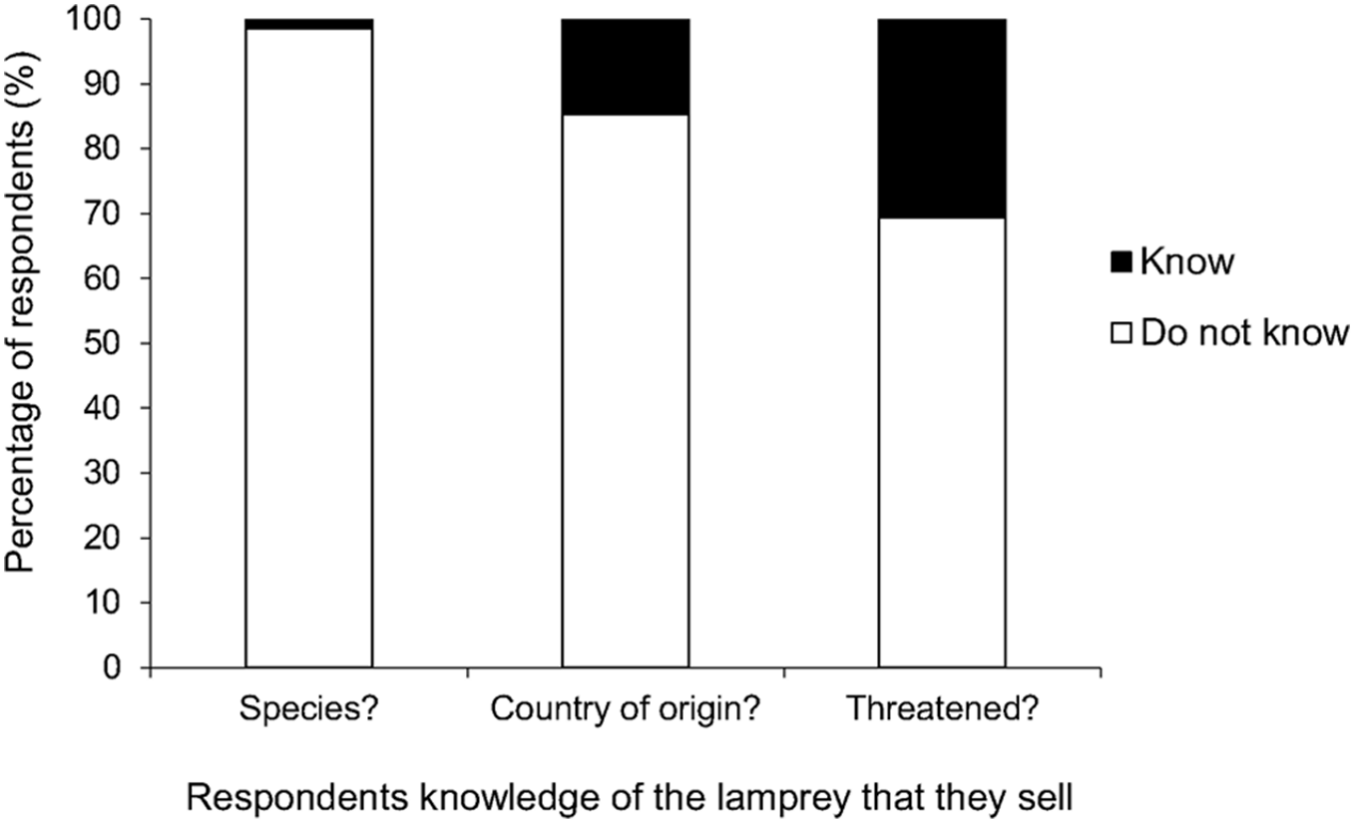
The percentage of tackle shop managers who know or do not know the species of lamprey that they sell, the country from which they originated, and whether they are from a threatened or non-threatened population.

Perhaps surprisingly, 98.5% of Supplier A (a direct supplier) respondents were unaware of which species of lamprey they sold, 85.3% were unaware of where they originated from, and 69.3% were unaware of whether they came from a threatened or non-threatened population (Fig. 4); 5.1% and 25.6% believed the lamprey they sold came from a threatened and non-threatened population, respectively. Only one respondent knew which river the lamprey that they sold originated from (River Trent, [Humber River Basin]), although five respondents said they believed the lamprey that they sold were from a sustainably farmed population (though no lamprey are farmed).

**Figure 5:**
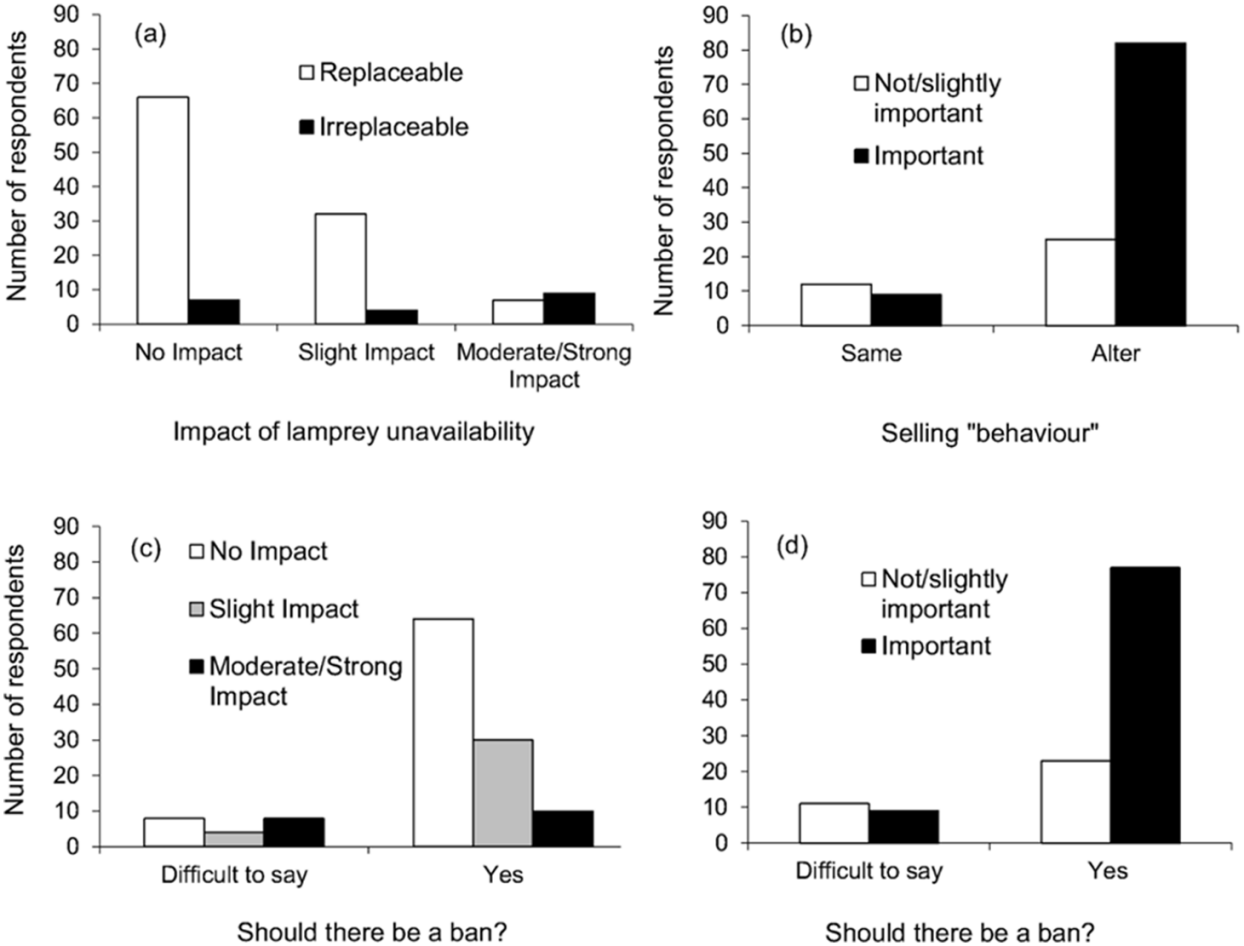
The distribution of tackle shop manager responses. regarding (a) the perceived ‘replaceability’ of lamprey vs the impact of lamprey unavailability on respondents’ businesses, (b) the importance of knowing if threatened or non-threatened vs decision to either sell the same amount, or alter (reduce/stop) their selling of, lamprey, (c) the impact of lamprey unavailability on respondents’ businesses, and (d) the importance of knowing if the lamprey they sell are threatened or non-threatened vs. respondents’ decision regarding a ban.

The majority of respondents (56.3%) believed lamprey unavailability would not impact their business, whilst 29.6%, 11.1% and 3.0% believed it would have a slight, moderate or strong impact, respectively. When asked if, in the event that lamprey were unavailable to sell, there would be other available products to sufficiently replace them, most (77.9%) said yes, 14.7% said no and 7.4% found it difficult to say. Baits most commonly given as suitable replacements for lamprey were native cyprinids, eel, smelt, mackerel and “bluey” (Pacific saury, *Cololabis saira*). No respondents suggested artificial lures, although they were not prompted to explain whether they felt artificial lures offered a suitable alternative to lamprey bait. There was no relationship between the number of lamprey stocked by respondents and the impact of lamprey unavailability (no impact/impact) on business (logistic regression: β = 0.001, SE = 0.001, d.f. = 1, *P* = 0.141). However, there was a highly significant association between the perceived ‘replaceability’ of lamprey (replaceable/irreplaceable) and the impact of lamprey unavailability on their business (*X* ^2^ = 22.16, d.f. = 2, *P*<0.001), with a significant number of respondents who claimed lamprey are an irreplaceable bait stating they would be impacted by lamprey unavailability (partial X^2^ = 16.20; Fig. 5a).

When asked how important it is to know whether the lamprey they sell originate from a threatened or non-threatened population, 71.0% of respondents said it was either very important or important, whilst 29.0% said it was slightly or not at all important. Only 16.2% of respondents would continue to sell the same amount of lamprey if they were reliably informed they were from a threatened population, whilst the majority of respondents said they would alter their selling “behaviour”, either by reducing the amount they sell (21.5%) or stopping the sales of lamprey altogether (62.3%). Furthermore, 77.0% of respondents said there should be a ban, whilst 8.2% said there should not be a ban and 14.8% found it difficult to say.

Respondents’ decision to alter their selling “behaviour” was significantly associated with how important they felt it is knowing if the lamprey they sell are threatened (*X* ^2^ = 9.35, d.f. = 1, *P* = 0.002; Fig. 5b). Respondents claiming it is slightly or not at all important decided they would keep selling the same amount of lamprey if they were informed they were threatened (partial *X* ^2^ = 4.86). The impact of lamprey unavailability on the respondents’ businesses, the number of lamprey stocked by respondents between 2011 and 2012 and the number of years over which respondents had been selling lamprey had no effect on their decision to alter their selling “behaviour”.

Whether respondents were in agreement or indecisive about a ban (too few disagreed for this to be incorporated into analysis) was highly dependent upon the impact of lamprey unavailability on their business (*X* ^2^ = 12.48, d.f. = 2, *P* = 0.001; Fig. 5.c) and how important they felt it is knowing if the lamprey they sell are threatened (*X* ^2^ = 8.02, d.f. = 1, *P* = 0.004; Fig. 5.d). Respondents whose businesses would be most impacted by lamprey unavailability, and those claiming it is slightly or not at all important knowing if the lamprey that they sell are threatened, were more indecisive over a ban than expected. The number of lamprey stocked by respondents and the number of years over which respondents had been selling lamprey had no effect on their decision regarding a ban (Table 2).

**Table 2:** The variables influencing respondents’ (Q2) decisions regarding their selling “behaviour” and a ban.

	Dependent variables	
Independent variables	Selling behaviour	Decision on Ban
Importance of knowing if threatened	√	√
Number of years selling lamprey	✗	✗
Number of lamprey stocked 2011–2012	✗	✗
Perceived ‘replaceability’ of lamprey	-	-
Impact of lamprey unavailability on business	✗	√

Ticks show a significant relationship between variables, crosses show no significant relationships were found, and dashes show that analyses could not be performed due to low response counts.

## Discussion

About 9 tonnes (over 90,000 lamprey) of river lamprey are supplied for angling use annually, almost exclusively, it would appear, in Britain, reflecting potential indirect effects of recreational fisheries on conservation species [28]. The bottom-up data, calculating lamprey supply to anglers (7.79–9.25 tonnes), overlap with the supplier figure, giving independent support for the scale of exploitation. Despite an initial expectation that most lamprey sold for bait in Britain would originate there, this proved not to be the case, with just 14% sourced from Britain. Supplier A has increasingly relied on lamprey from Estonia since 2002 and Supplier C has been importing lamprey from The Netherlands since *c.* 2005. Hence, the proportion of lamprey originating from Britain has declined markedly since the lamprey bait trade started in 1995 (when all lamprey were sourced from Britain) and before the fishing regulations in Britain were imposed in 2011. However, there are no official statistics or records of this trade in exporting or importing countries.

### 4.1 Commercial Catches in the Ouse

Our findings confirm that Masters et al. [25] were correct in suggesting that the exploitation of river lamprey in the tidal Ouse may have been twice the level they calculated (12.0% after accounting for mark loss of tagged lamprey at liberty). Before catch restrictions were imposed in 2011, Fisher B’s total catches were 74.1–108.4% of Fisher A’s total catches for the same seasons, suggesting that Fisher B may have always operated at a similar scale to Fisher A since lamprey fisheries established. Furthermore, a third fisher had, in the recent past, been operating in the Ouse, taking 400–500 kg of river lamprey per season, but has now retired (Anon. pers. comm.). Hence, up until the end of the 2009 fishing season, a more realistic exploitation level in the tidal Ouse was >20%. This mortality level is substantial since river lamprey is a semelparous species which, by its life history, is susceptible to large-scale exploitation [42].

Despite this, analysis of CPUE data gave no indication that the Ouse lamprey stock had declined between 2000 and 2012. However, detecting a meaningful decline in mean seasonal CPUE is difficult over a relatively short time period, even though it incorporates year classes spawned just before the fishery’s onset, as well as since. It is also possible that the Ouse fishery could be acting as a population sink, as anadromous lampreys tend to disperse widely and are not philopatric [43]. This appears to be so for European river lamprey in the north western part of its range (F. Bracken, unpublished data), so commercial fishing in the Ouse might contribute to an overall decline in the population which is not detected in that single watershed. Migration of river lamprey into spawning rivers may rely on pheromone attraction [44] and this could facilitate population source to sink movements. Other studies have been able to collate and analyse extensive datasets of lamprey catches, spanning a century in one case [13], and although catches or CPUE differed markedly between years in these studies, the authors were able to distinguish long term trends [16], [17]. Therefore, our analysis should be treated with care, and more years of data, and perhaps wider information on lamprey population trends from other rivers within and outside Britain, will be required to support or refute our findings.

### 4.2 Regulation and Sustainability of Lamprey as Bait

The UK Marine and Coastal Access Act 2009 has aided regulation of lamprey exploitation in Britain, and has proved sensible given the market force demonstrated. Authorising the trapping of lamprey now allows close monitoring of exploitation levels through the obligatory provision of catch returns. Furthermore, since 2011, a maximum of 1 044 kg and 206 kg of river lamprey can be taken from the Ouse and Trent per fishing season, respectively, between 1^st^ November and 10^th^ December. This represents an estimated 5% average exploitation impact on each stock. The peak in CPUE estimated in this study falls within these dates, and on average 68% of all catch in the Ouse is made between 8^th^ November and 7–9^th^ of January, therefore the catch limit imposed is a crucial component in regulating exploitation. Consequently, whilst it is important to ensure there is no illegal trapping outside these dates, catch limits must be enforced during this period when the threat of overexploitation is highest. Fishery-independent CPUE data also needs to be collected outside the current fishing period to allow comparison with periods before regulations were imposed. Active management of this type, along with continued dialogue with lamprey fishers, is necessary to ensure the sustainability of lamprey in the Humber River Basin.

In this study smelt, a threatened species in Britain [45], were the most popular fish bait in 11.4% of tackle shops surveyed. Given that current national legislation applies for smelt as well as river lamprey, there should be careful monitoring of smelt exploitation in Britain which may rise as a result of lamprey fishery restrictions; smelt were commonly cited by tackle shop managers as a substitute bait for river lamprey. European eel, a species listed as ‘critically endangered’ globally [46] were also considered by some tackle shop managers to be a suitable substitute bait. However, although eel recruitment has declined by 90% across most of Europe [47], they remain far more widespread than river lamprey, and whilst river lamprey remain rare in most British rivers, it has been suggested that eel stocks in some, perhaps many, rivers along the west coast of England and Wales, and possibly some rivers in north-east England, are still at or near to carrying capacity [48]. It is interesting to note, therefore, that the Pike Anglers Club of Great Britain currently discourages members from using European eel as pike bait, but does not discourage the use of lamprey (http://www.pikeanglersclub.co.uk/the-use-of-eels-as-bait-guidance/). Along with our findings that almost 70% of tackle shop managers that were contacted in this study were unaware of whether the lamprey they sold were threatened, there is likely a wider lack of awareness of the conservation status of species used as bait.

The lamprey bait market in Britain is now mainly reliant upon river lamprey stocks in continental Europe, with only ∼14% of lamprey being sourced from Britain (Humber River Basin) in 2011–2012. We estimated that a maximum of 6.1 tonnes of lamprey were sourced from The Netherlands between 2011–2012, of which 3 tonnes were by-catch in eel fisheries there, and 1.6 tonnes were sourced from Estonia over the same period. FAO [49] statistics suggest that 59 tonnes of river lamprey were captured in Estonia in 2009, and the highest recent catch was 67 tonnes in 2008. Although river lamprey catches have declined in Estonia over the last 70 years, probably due to loss of spawning grounds [50], the stock is “generally stable in the rivers of Estonia” (Estonian Fisheries Strategy 2007–2013 [51]). Jansen et al. [52] state that river lamprey are common in The Netherlands and found in all major flowing waters, particularly the rivers Meuse and Rhine. Typically, a few thousand lamprey are caught per year in total recorded in logbook schemes, and the population in all rivers is considered to be in the 100 000s [52]. However, river lamprey is listed as vulnerable in the Dutch Red List [53], [54] and up to 6.1 tonnes (over 60,000) of lamprey were sourced from waters in The Netherlands in 2011–2012. This represents a substantial exploitation rate based upon the population estimates by Jansen et al. [52], and the bait market in Britain is likely having a significant impact on river lamprey population(s) in The Netherlands. There appear to be no requirements for formal records of lamprey landings and exports in The Netherlands, but legislation to restrict fishing seasons for river lamprey was introduced in 2012 under the Visserijwet (Fisheries Act); fishermen can only catch lamprey outside of the main migration period (1^st^ November–31^st^ January) and spawning period (1^st^ March–31^st^ April; H. Winter, pers. comm.).

While regulation of commercial fishing for lamprey in Britain has facilitated improved sustainability in lamprey populations there, continued demand is currently being met mostly from imports. As well as conservation risks for lamprey in those countries (if populations and exploitation rate are unknown) this brings additional risks, notably the potential for transfer of virus-borne diseases by frozen bait, from localities of origin to sites where these diseases do not occur [55]. Such disease risks are currently poorly known for lamprey and for other dead fish baits, such as European eel and cyprinids, but it is known that freezing is ineffective in inactivating viruses in dead baitfish [55]. In this respect, home-caught lamprey represent less of a risk than imported ones. Low-impact, carefully regulated bait fisheries, in this case for lamprey, within the country of use, may also offer useful CPUE information for conservation support.

### 4.3 Stakeholder Knowledge and Impacts of Catch/Sale Restrictions

Studies have often highlighted differences within and between stakeholder groups in terms of their knowledge and attitudes, and stress how these differences need to be recognised by conservationists when designing and implementing management policies [31], [56]–[60]. Knowledge of wholesaler and shop manager stakeholders in relation to the lamprey that they sold varied greatly. There was a lack of awareness amongst tackle shop managers about which species of lamprey they sold and where they originated from; just 1.5% of managers knew they sold river lamprey and only 14.7% of managers could provide the country from which they believed they were sourced. In comparison, the main wholesale supplier managers were relatively knowledgeable about the lamprey that they sold, with all suppliers knowing the country or origin and all but one knowing which species they sold. Despite the tackle shop managers’ lack of knowledge towards the lamprey they sold, the vast majority were positive towards the regulation of their sales. For instance, 83.8% of managers said they would either reduce or stop selling lamprey if they were reliably informed they were threatened, although slightly fewer managers (77.0%) were prepared to support a ban on the capture and selling of lamprey in Britain if they were considered to be threatened. Similarly, most wholesale suppliers stated they would discontinue lamprey sales if they were reliably informed they were threatened. However, suppliers were mostly indecisive towards a ban, as three of the five suppliers said they would either be moderately/strongly impacted by lamprey unavailability and all but one felt that lamprey are an irreplaceable product. Most suppliers would need to be convinced that the lamprey they supply are under threat before they would support a ban. This underscores how essential it is to communicate with key stakeholders during the development of management policies to anticipate any negative impacts on their businesses [27], [31], [32]. Wholesale supplier managers in this case represent the stakeholders who would, in general, be most affected by regulations in lamprey fisheries either in Britain or elsewhere in Europe.

The tackle shop managers who would not personally alter their sales were those who felt it was unimportant knowing if the lamprey they sell are threatened. This apparent lack of conservation concern was also associated with indecisiveness over a ban, although managers whose businesses would be most impacted by lamprey unavailability were also those who were indecisive over a ban. This suggests that, regardless of whether managers’ businesses would be impacted by lamprey unavailability, most would personally alter their selling behaviour, although when it came to strict state regulation (i.e. ban) the impact of lamprey unavailability became a determining factor when deciding whether to agree to a ban. Similarly, Dorow et al. [26] detailed how eel anglers were willing to accept tight regulations on recreational eel harvestings (e.g. reduction in daily bag limit), although were strongly against any form of temporal closures to the fisheries. Dorow et al. [31] indicated that advanced eel anglers would be most affected by strict regulations because it would be difficult for them to find another acceptable fish species or recreational activity to substitute for eel fishing. Interesting parallels can be drawn between Dorow et al. [26] and this study, as those tackle shops impacted the most by lamprey unavailability were those that considered lamprey to be an irreplaceable product. Many tackle shop managers considered that the durability and high blood content and the scent trail that lamprey leave in the water makes it very effective as pike bait.

In the near future, it would be prudent to evaluate the knowledge and attitudes of predator (especially pike) anglers in Britain using lamprey as bait. It would be important to establish whether anglers feel there are adequate substitutes to river lamprey (e.g. other natural baits or artificial lures) when fishing for pike, given that most managers (77.9%) in this study felt there were. A study evaluating the effectiveness of different “baits” for pike (including natural baits and artificial lures – spinners, spoons, plugs and soft plastic baits) demonstrated that the size of pike caught was mostly related to bait size rather than bait type [61]. Furthermore, Arlinghaus et al. [61] showed that natural baits were swallowed more deeply than artificial baits that may lead to hooking mortality. It appears, therefore, that there are alternatives to using lamprey when fishing for pike, although the effectiveness of baits likely varies between waters and some anglers may exhibit strong preferences for bait types.

### 4.4 Conclusions

Currently it appears that top-down legislative regulation has provided the means for better protection of river lamprey in Britain from exploitation, but market forces have altered the source of lamprey supply, a pattern frequent in the wildlife trade [8], [62]. Although river lamprey are not sufficiently threatened to be afforded CITES-type trade protection, it is surprising that there are no formal data on imports and exports. This study exemplifies how simple but poorly known markets can drive international, largely unregulated, supply of conservation species, often to the ignorance of regulatory authorities, even in developed countries with strong environmental and wildlife regulatory resources. In such a context, the difficulties faced by developing nations managing wildlife exploitation and trade deserve fuller appreciation. Longer term effective conservation of lamprey bait species (or alternative species such as smelt) will require agreements from all stakeholders, including anglers, as well as international cooperation [30], [32]. It may also necessitate increased biosecurity. Introduction of a system of eco-certification for the sourcing of lamprey by wholesalers as being from sustainable sources, similar to (or part of) that used by the Marine Stewardship Council (http://www.msc.org) for sustainability of seafood, could be a useful measure. Given that this study demonstrates the paradoxical exploitation of a conservation species of fish by anglers who mostly take great care to ensure the careful return of their quarry (http://pikeanglersclub.co.uk/pike-conservation/), education and discussion may prove to be the most effective tool for reducing and ensuring the sustainability of lamprey for angling in the longer term.
